# Supplementary material for: Synthesis and Functionalisation of Superparamagnetic Nano-Rods towards the Treatment of Glioblastoma Brain Tumours
Source: Nanomaterials (Basel). 2021 Aug 24;11(9):2157. doi: 10.3390/nano11092157 (PMC8472662; doi:10.3390/nano11092157)
Supplement: Supplementary file 1 [file nanomaterials-11-02157-s001.zip › nanomaterials-1279394-SI.pdf]

# Supplementary Materials

# Synthesis and Functionalisation of Superparamagnetic Nano-Rods towards the Treatment of Glioblastoma Brain Tumours

**Kinana Habra [1], Stéphanie E. B. McArdle [2], Robert H. Morris [1] and Gareth W. V. Cave [1,*]**

[1] School of Science and Technology, Nottingham Trent University, Nottingham NG11 8NS, UK; kinana.habra2017@my.ntu.ac.uk (K.H.); rob.morris@ntu.ac.uk (R.H.M.)
[2] John van Geest Cancer Research Centre, School of Science and Technology, Nottingham Trent University, Nottingham NG11 8NS, UK; stephanie.mcardle@ntu.ac.uk
* Correspondence: gareth.cave@ntu.ac.uk; Tel.: +44-115-84-83242

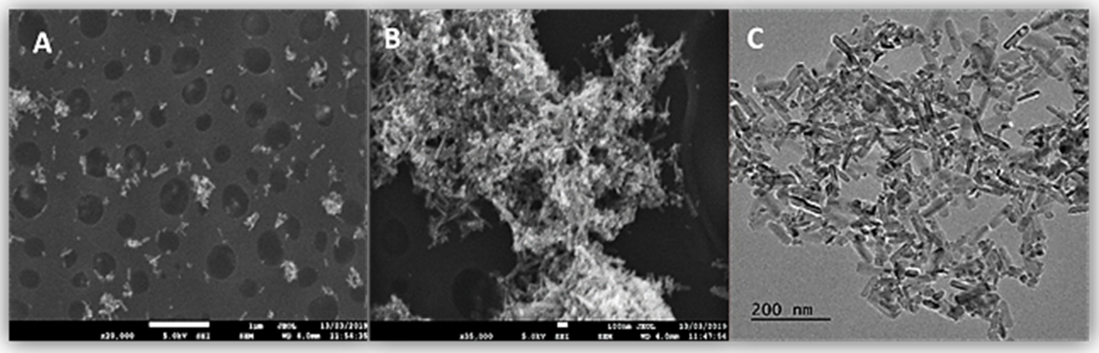


**Figure S1** (A, B) SEM images show the aggregates of naked iron oxide nano-rods before coating. The scale bars are 1 µm, 100 nm. (C) The TEM image shows similar aggregates for uncoated IONRs.

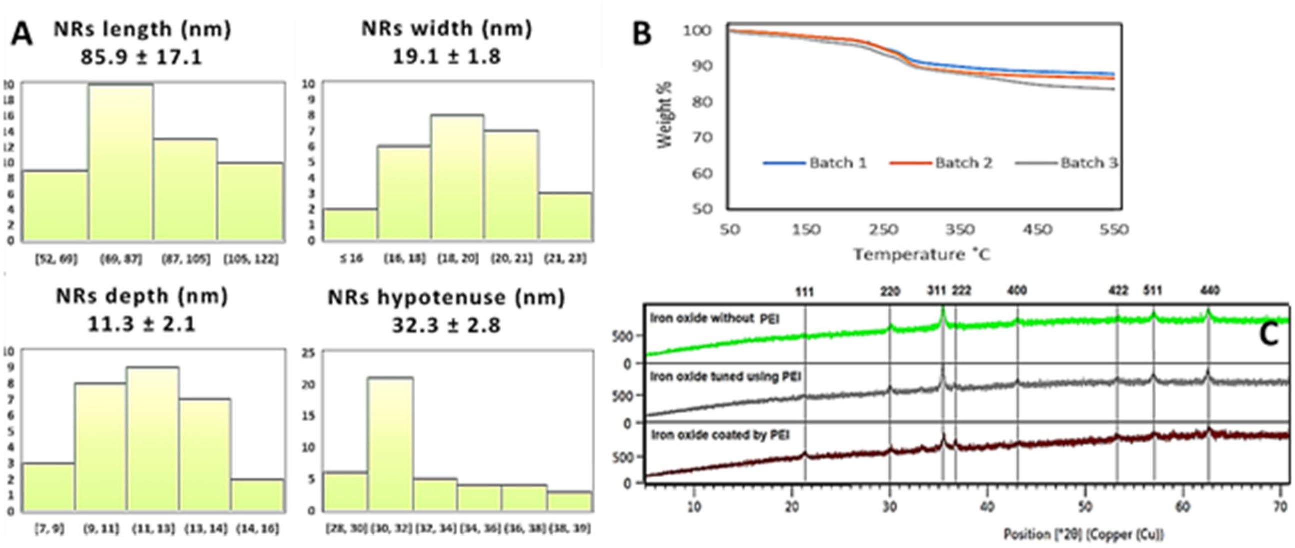


**Figure S2** (A) Image J analysis for the dimension distribution for the tetragonal prism-dipyramid morphology of the iron oxide nano-rods (length, width, depth, and hypotenuse). (B) Thermogravimetric analysis (TGA) shows the repeatability of the coating method for three batches. (C) X-ray diffractogram (XRD) displays the labelled characteristic planes (111, 220, 311, 222, 400, 511, 440) for the iron oxide crystal pattern (naked, tuned with polymer and coated with extra layer of polymer).

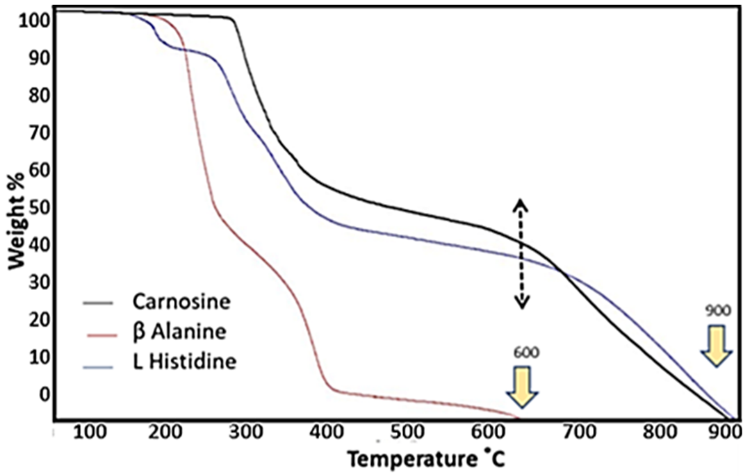


**Figure S3** Thermogravimetric analysis (TGA) shows the carnosine compounds. Carnosine shows a distinct two stage weight loss with an onset at 196 °C. An initial weight loss of 46.04% and a second weight loss of 45.79% equate to a total mass loss of 91.83%. The major loss of β-Alanine occurs between 200 and 300 °C. L-Histidine is the responsible for the loss pattern between 600 and 900 °C.

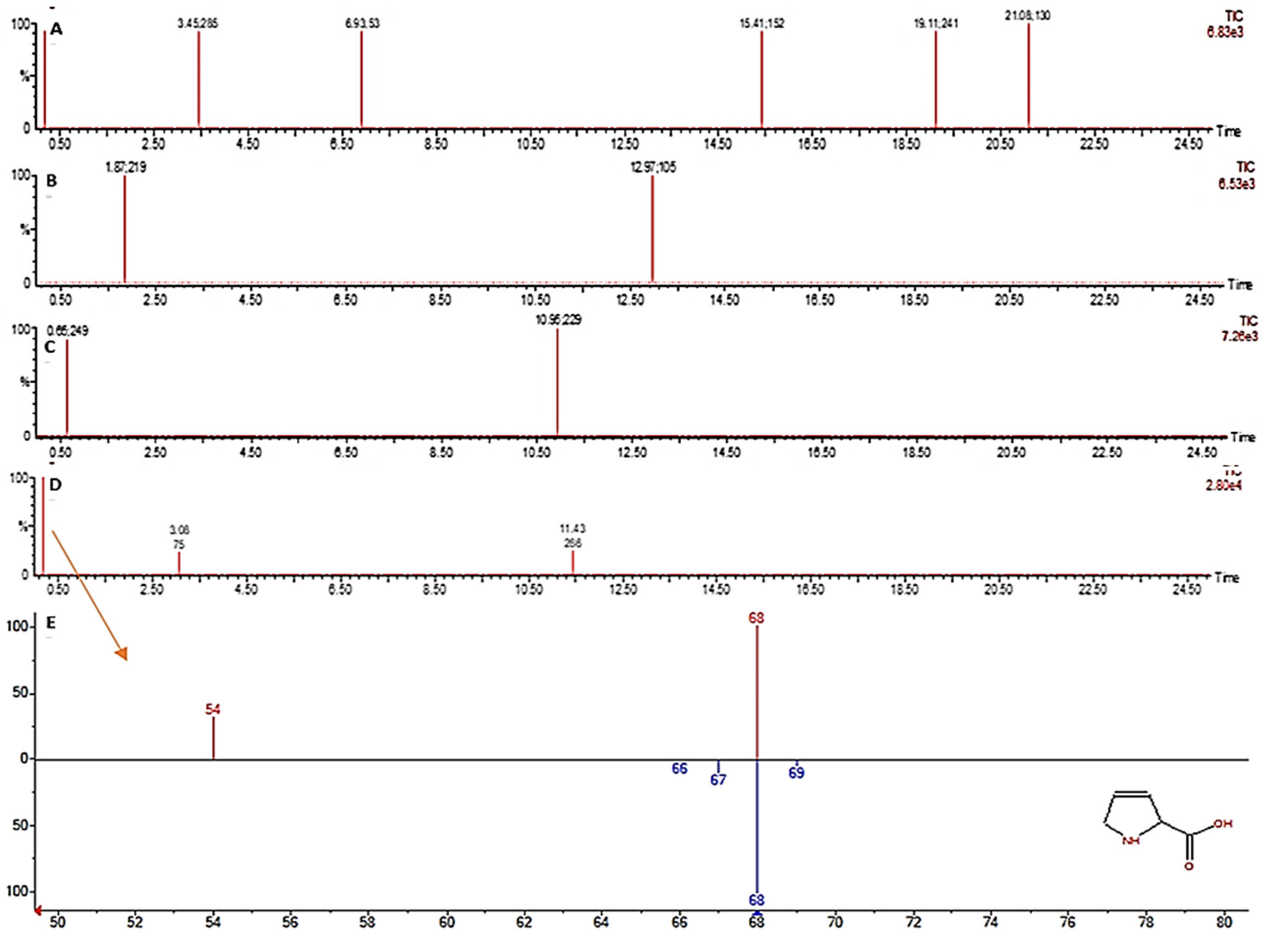

**Figure S4 GC-**MS spectrums analyse the organic coat on the IONRs which evaporate at (A) 13min, (B) 22min and (C) 25min, which cover the range between 150 and 600 °C. (D,E) Specific peak for carnosine/PEI coated IONRs between 600 and 800 °C matches imidazole in MS library from L-Histidine.

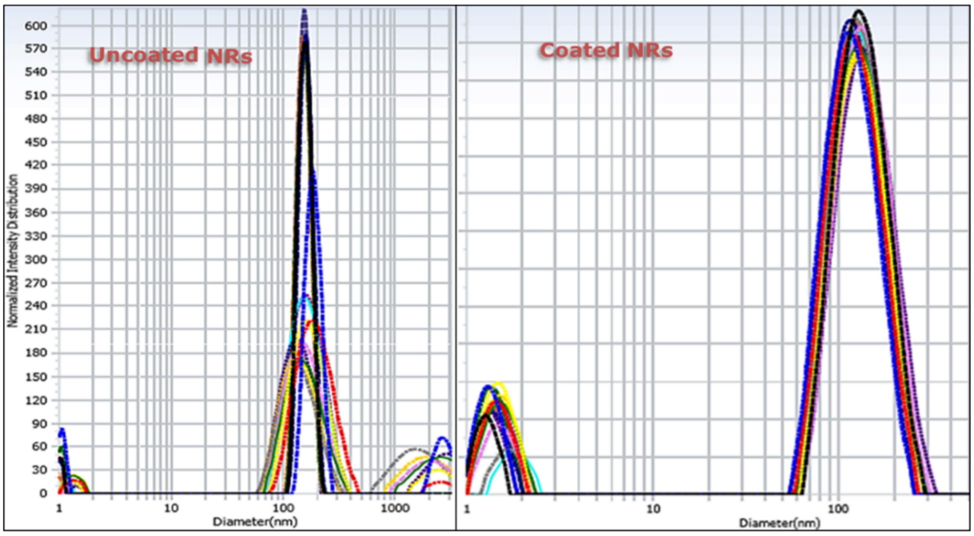


**Figure S5** Dynamic light scattering DLS compering the intensity distribution of the iron oxide nano-rods' hydrodynamic diameter before and after coating. The polymer coating prevents the aggregation. After coating, the rods had a relatively stable hydrodynamic diameter over the course of an hour. Each measurement is the mean of 60 measurements repeated 3 times over 3 min to reduce the error to the minimum.

| 1 hour | Uncoated IONRs | BPEI coated IONRs, pH7 | BPEI coated IONRs, pH3 |
|---|---|---|---|
| Hydrodynamic diameter | 0.3111 | 0.254 | 0.1228 |
| Diameter polydispersity | 0.6528 | 0.2768 | 0.0125 |

**Table S1** R2 for the Hydrodynamic diameter and polydispersity of the IONRs at different conditions over 1 h.

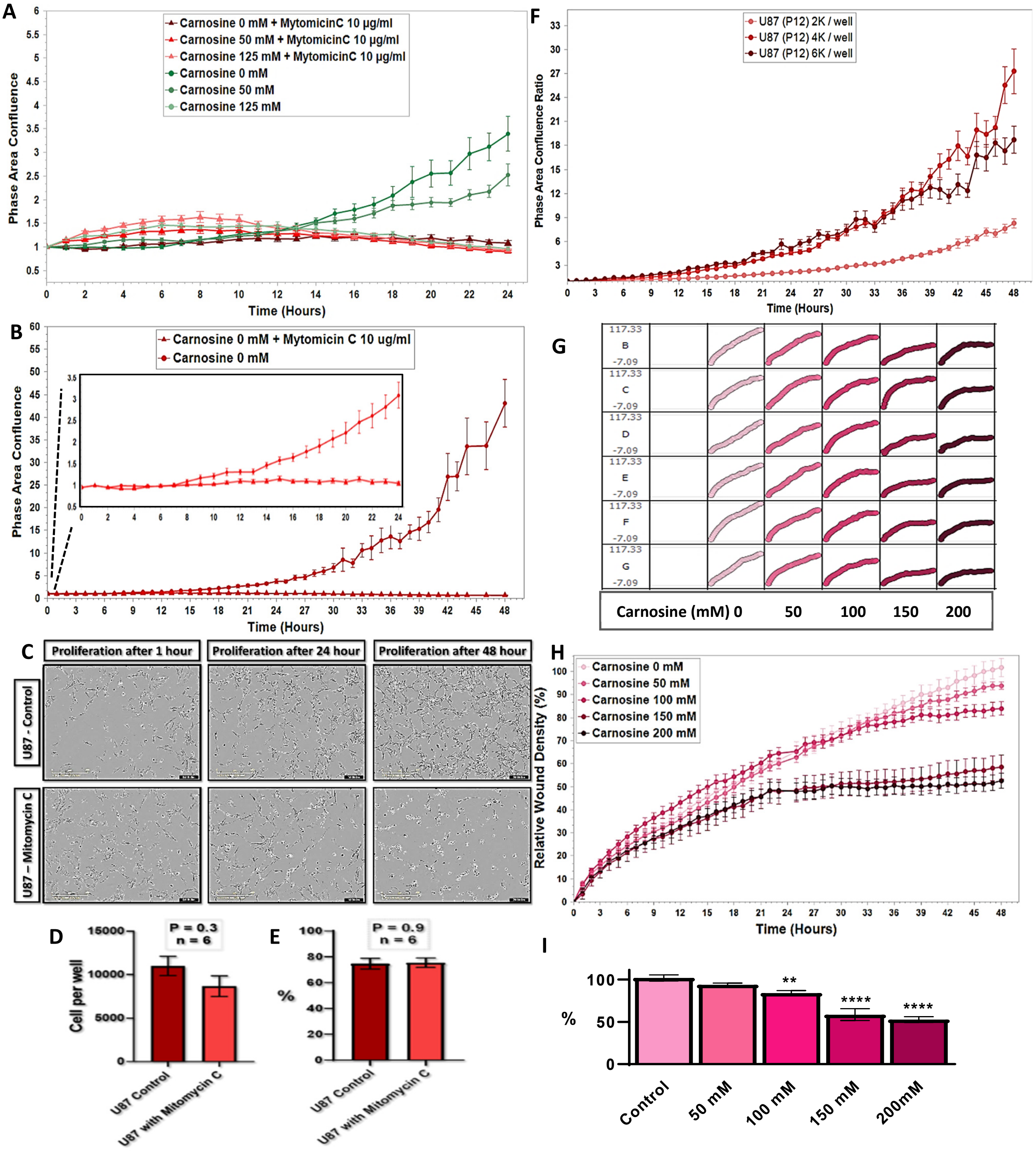


**Figure S6** IncuCyte system images analysis for U87 cells' confluence phase shows: (A) the effect of carnosine on the cells was not affected by using mitomycin C (B) the difference between the normal increase in proliferation for the cells and the effect of adding mitomycin C to stop the proliferation. (B) Phase confluence proves the continuous splitting for control cells. However, the cells with mitomycin C stop splitting and start dying naturally after 24 h. Scale bars are 400 µm. (C) Images of the cells in control sample and with mitomycin C at (1, 24, 48) hour. (D) The number of the adherent cells with and without the addition of mitomycin C. (E) The percentage of the viable cells with and without adding mitomycin C. (F) proliferation curves of the optimising the confluence ratio of U87 cells upon carnosine exposure over 48 h (4000 cell/well is the best). (G) The matrix of relative wound density for 6 samples of each concentration (0, 50, 100, 150 µM, respectively) over 2 days. (H) The effect of different carnosine concentrations added to U87 cells after 18 h of adherence when the scratch was applied. The scratch wound starts healing by proliferation and migration. Cells were imaged in 96 well plates while growing in media with different carnosine treatments using IncuCyte S3 live cell imaging system to take two snaps per well each hour over 2 days. The phase confluence was analysed by the standard IncuCyte software, and the relative wound density was analysed by the integrated IncuCyte wound scratch software and plotted against elapsed time in hours. A time of 0 h indicates the cell density 30 mins after placing the plate in the IncuCyte. All cells' variations in growth arised after approximately 8 h. The supportive movies of cell migration are available in the supplementary movies. (I) The statistical analysis shows the significant effect starts between 50 and 100 mM of carnosine for a cell density of 103 per well. The statistical significance level indicated with (**) for $p < 0.01$, and (****) for $p < 0.0001$.

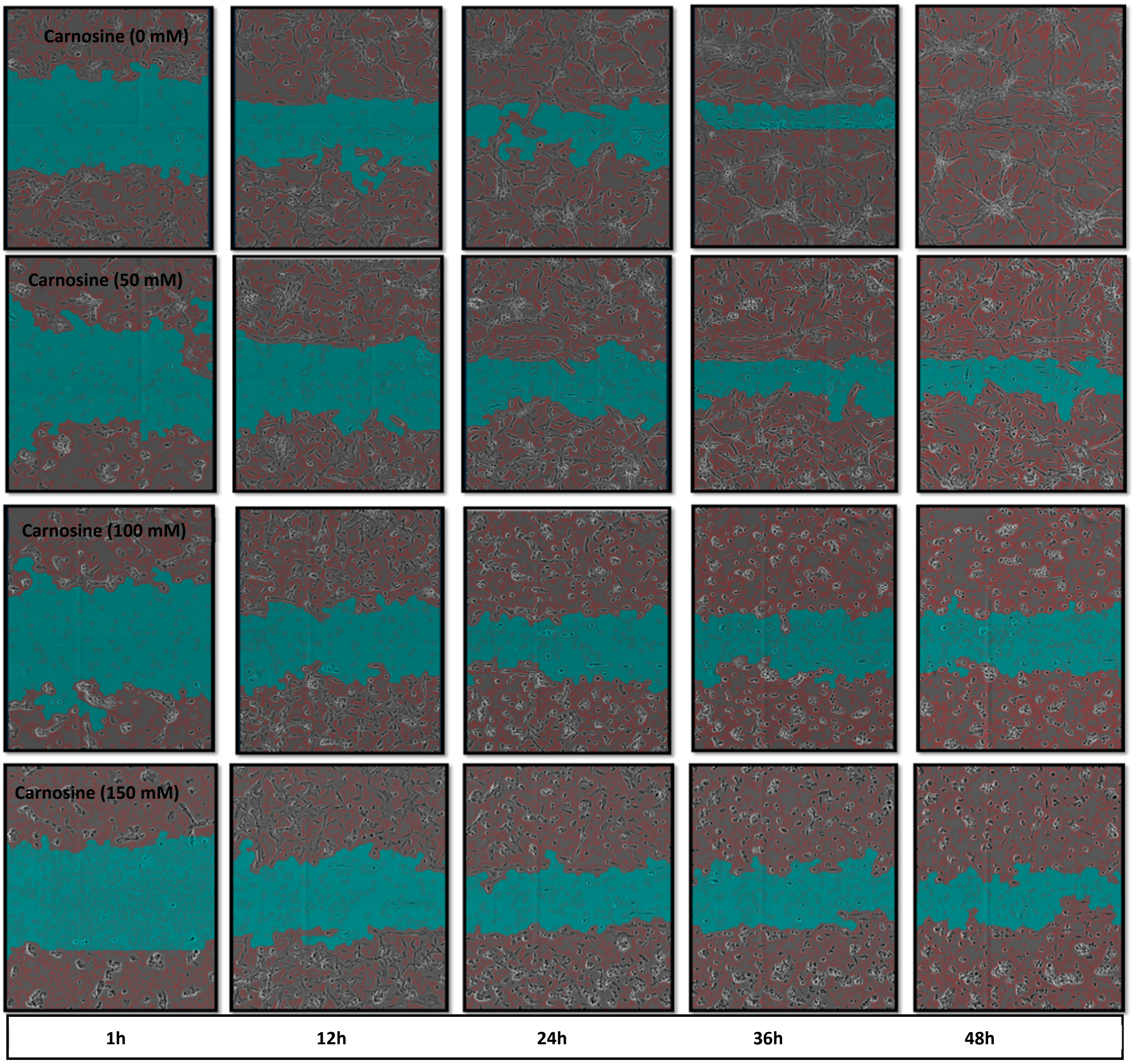


**Figure S7** Phase images with mask show the effect of different IONRs' concentrations on U87 cells' wound healing by proliferation and migration. Cells were imaged in 96 well plate while growing in media with different carnosine treatments using IncuCyte S3 live cell imaging system each 12 h for 2 days

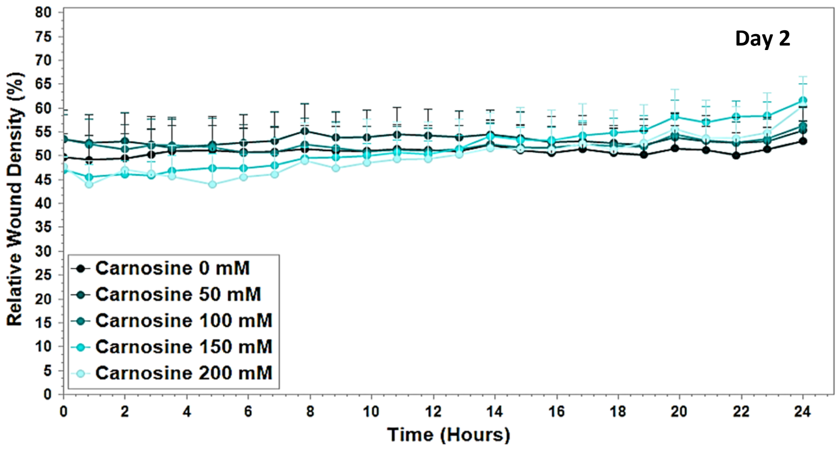


**Figure S8** Effect of different IONRs' concentrations on U87 cells' wound healing by migration after ceasing the proliferation with mitomycin C. Cells were imaged in a 96 well plate while growing in media with different carnosine treatments using the IncuCyte S3 live cell imaging system to take two snaps per well each hour. The migration stopped after 24 h due to cell death instead of splitting, and the figure showed a plateau

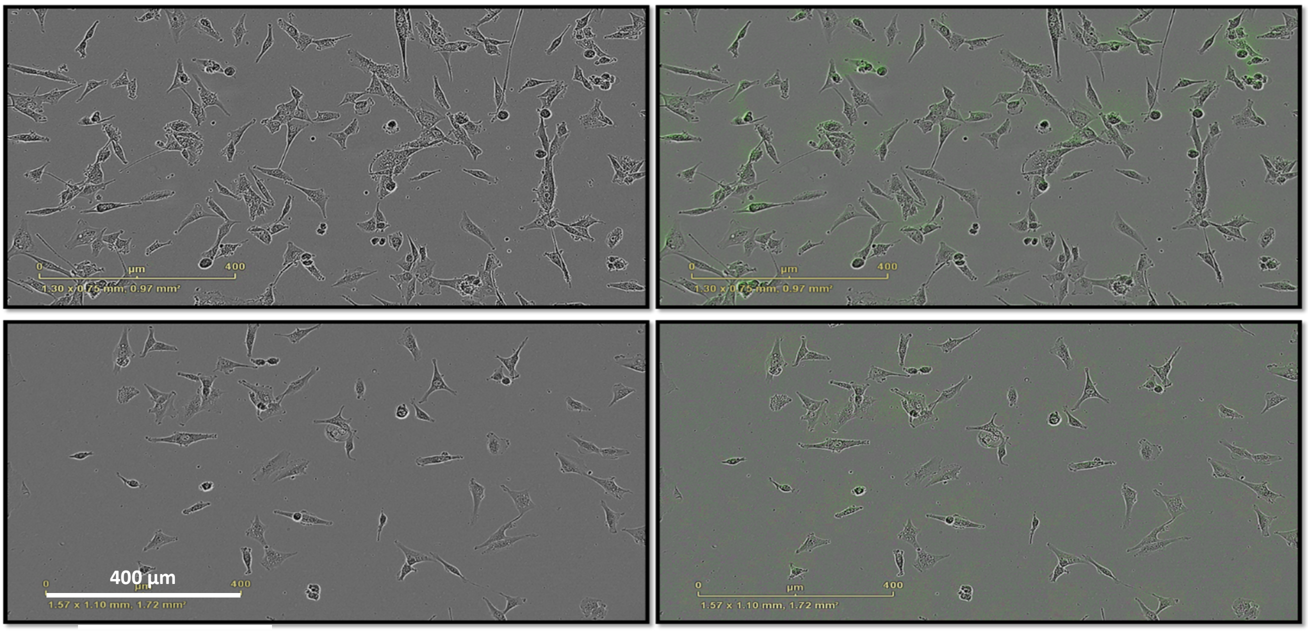


**Figure S9** IncuCyte S3 live cell system (10×) images show proliferation of U87 cells upon carnosine exposure after 24 h. Cells were compared with and without treatment of 100 mM carnosine concentration in the presence of CytoLight Rapid Dye for live-cell green labelling dye (200 nM) which enters live cells' membranes and emits green fluorescence. Each photo was displayed as phase and green channel without mask. A decrease in the cells' population appeared after 24 h. However, the cells with no morphology change are the daughters who stay alive. Scale bars are 400 µm.

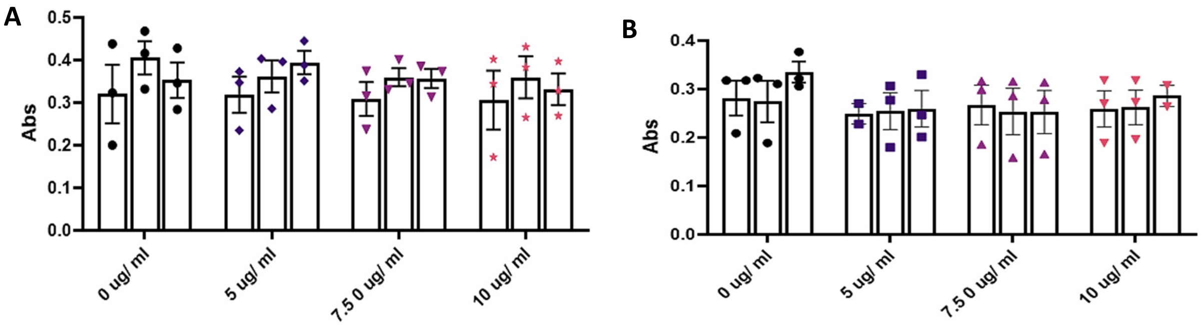


**Figure S10** MTT cell viability assay on U87 cell line (A. mitotic vs. B. differentiated) with varying concentrations of BPEI-coated IONRs after 48 h. Error bars represent the standard error of the mean (SEM). The symbols show the distribution of the results for different passages.

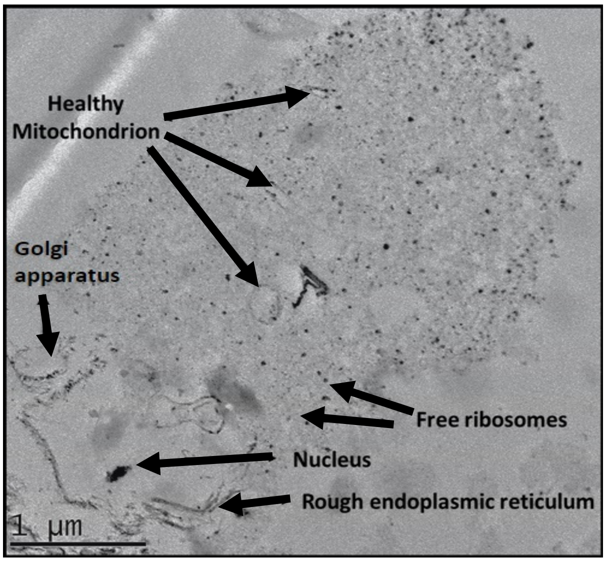


**Figure S11** TEM images of U87 cell sections without any treatment.

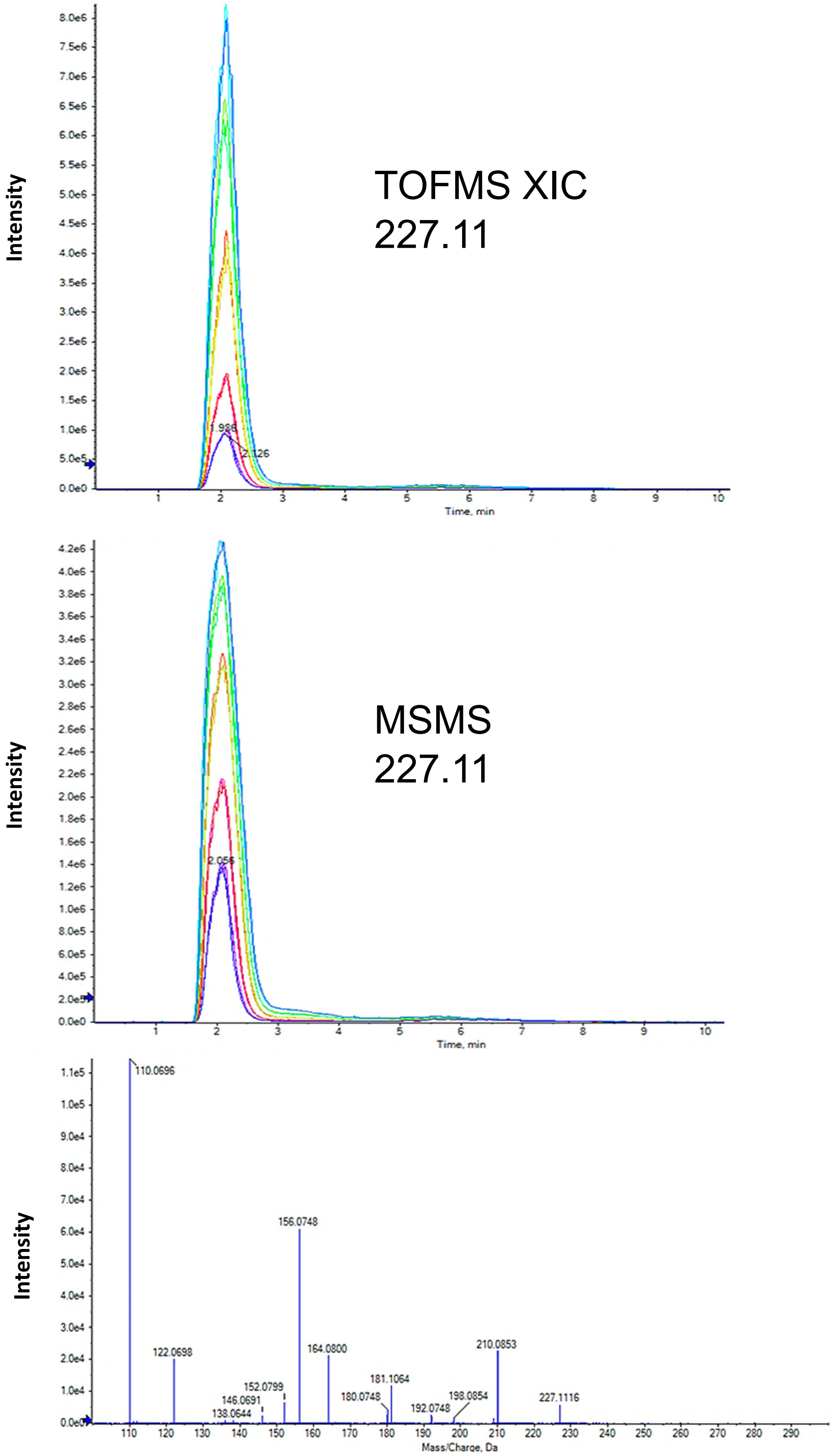


**Figure S12** LC-Ms chromatograms for the standards and samples from the tubing experiment. All appeared at the same retention time and showed the same characteristic spectrum of carnosine.
